# Neutrophil extracellular traps characterize caseating granulomas

**DOI:** 10.1038/s41419-024-06892-3

**Published:** 2024-07-31

**Authors:** Leticija Zlatar, Jasmin Knopf, Jeeshan Singh, Han Wang, Marco Muñoz-Becerra, Irmgard Herrmann, Rebecca C. Chukwuanukwu, Markus Eckstein, Philip Eichhorn, Ralf J. Rieker, Elisabeth Naschberger, Andreas Burkovski, Veit Krenn, Rostyslav Bilyy, Tetiana Butova, Iryna Liskina, Ihor Kalabukha, Oleg Khmel, Michael Boettcher, Georg Schett, Dmytro Butov, Anton Tkachenko, Martin Herrmann

**Affiliations:** 1 Department of Internal Medicine 3—Rheumatology and Immunology, Friedrich-Alexander-University Erlangen-Nürnberg and Universitätsklinikum Erlangen, Erlangen, Germany; 2 Deutsches Zentrum für Immuntherapie (DZI), Friedrich-Alexander-University Erlangen-Nürnberg and Universitätsklinikum Erlangen, Erlangen, Germany; 3 Department of Pediatric Surgery, University Medical Center Mannheim, University of Heidelberg, Mannheim, Germany; 4 Immunology Unit, Medical Laboratory Science Department, Faculty of Health Sciences, Nnamdi Azikiwe University, Awka, Nigeria; 5 CCC Comprehensive Cancer Center (CCC) Erlangen and Institute of Pathology, Friedrich-Alexander-University Erlangen-Nürnberg, Erlangen, Germany; 6 Division of Molecular and Experimental Surgery, Universitätsklinikum Erlangen, Friedrich-Alexander Universtität Erlangen-Nürnberg, Erlangen, Germany; 7 Microbiology Division, Department of Biology, Friedrich-Alexander-University Erlangen-Nürnberg, Erlangen, Germany; 8 MVZ-Center for Histology, Cytology and Molecular Diagnostics, Trier, Germany; 9 Lectinotest R&D, Lviv, Ukraine; 10 Outpatient Department, Merefa District Hospital, Merefa, Ukraine; 11 Department of Pathomorphology, State Organization “National Institute of Phthisiology and Pulmonology named after F.G. Yanovsky of the National Academy of Medical Sciences of Ukraine”, Kyiv, Ukraine; 12 Department of Surgical Treatment of Tuberculosis and Non-Specific Lung Diseases, State Organization “National Institute of Phthisiology and Pulmonology named after F.G. Yanovsky of the National Academy of Medical Sciences of Ukraine”, Kyiv, Ukraine; 13 Department of Infectious Diseases and Phthisiology, Kharkiv National Medical University, Kharkiv, Ukraine; 14 Research Institute of Experimental and Clinical Medicine, Kharkiv National Medical University, Kharkiv, Ukraine; 15 BIOCEV, First Faculty of Medicine, Charles University, Vestec, Czech Republic; 16 FAU Profile Center Immunomedicine (FAU I-MED), Friedrich-Alexander-Universität (FAU) Erlangen-Nürnberg, Erlangen, Germany

**Keywords:** Tuberculosis, Prognostic markers, Neutrophils, Immune cell death

## Abstract

Tuberculosis (TB) remains one of the top 10 causes of death worldwide and still poses a serious challenge to public health. Recent attention to neutrophils has uncovered unexplored areas demanding further investigation. Therefore, the aim of this study was to determine neutrophil activation and circulatory neutrophil extracellular trap (NET) formation in various types of TB. Sera from TB patients (*n* = 91) and healthy controls (NHD; *n* = 38) were analyzed for NE-DNA and MPO–DNA complexes, cell-free DNA (cfDNA), and protease activity (elastase). We show that these NET parameters were increased in TB sera. Importantly, NET formation and NE activity were elevated in TB patients with extensive tissue damage when compared to those with minor damage and in patients with relapse, compared to new cases. We discuss the importance of balancing NET formation to prevent tissue damage or even relapse and argue to analyze circulating NET parameters to monitor the risk of disease relapse. To investigate the tissues for NETs and to find the source of the circulating NET degradation products, we collected sections of granulomas in lung and lymph node biopsies. Samples from other diseases with granulomas, including sarcoidosis (SARC) and apical periodontitis (AP), served as controls. Whereas NET formation characterizes the caseating granulomas, both caseating and non-caseating granulomas harbor DNA with unusual conformation. As TB is associated with hypercoagulation and thromboembolism, we further imaged the pulmonary vessels of TB patients and detected vascular occlusions with neutrophil aggregates. This highlights the dual role of neutrophils in the pathology of TB.

## Introduction

Tuberculosis (TB), an infectious disease caused by airborne transmission of *Mycobacterium tuberculosis* (MTB), manifests itself through fever, coughing, and severe chest pain [1]. TB remains a significant global health concern, particularly in low- and middle-income countries, as a leading cause of death among all infectious diseases [2, 3]. Drug-resistant strains pose a major challenge to TB control efforts [4, 5]. These include monoresistant TB, multi-drug-resistant TB (MDR TB), pre-extensively drug-resistant TB (Pre-XDR-TB), and extensively drug-resistant TB (XDR-TB). The ability of MTB to disseminate via the bloodstream and lymphatic system can further lead to extrapulmonary TB [6]; commonly affecting the lymph nodes, skeleton, genitourinary system, central nervous system and others [7].

In response to MTB infection, various immune cells are involved [8]. Their aim is to overcome the pathogen’s evolutionary evolved immunity-escaping pathways [9, 10]. In humans, neutrophils are the predominant immune cell type in MTB-infected lung during active TB [8, 11]. In the early phases of infection, large numbers of neutrophils migrate into the lungs and, along with macrophages, promote the elimination of the pathogen by phagocytosis [12–14]. However, MTB can survive within neutrophils [15] or induce neutrophil necrosis. The uptake of infected neutrophils by macrophages promotes MTB growth [16]. Neutrophils form neutrophil extracellular traps (NETs) in response to bacterial infections [17–19], also in TB [13]. These structures contain DNA, histones and antimicrobial proteins such as neutrophil elastase (NE) and myeloperoxidase (MPO) [20]. Importantly, NET-bound serine proteases escape the control by their natural antagonists, the serpins. Consequently, NETs harbor a substantial proteolytic activity able to degrade not only pro-inflammatory cytokines [21] but also tissue components like the extracellular matrix. They were shown to isolate areas of necrotic tissues in pancreatitis and other massive tissue damage [22]. NET formation upon neutrophil activation by MTB was initially thought to capture and destroy the pathogen [8, 12, 23, 24]. However, NETs may not effectively kill MTB [12, 13, 23]. NET formation in the early phases of TB may trap bacteria and prevent their spread, aiding macrophages in MTB engulfment and destruction [11, 13]. MTB-activated neutrophils closely interact with macrophages, which may bind to and phagocytose NETs [11, 12]. The clearance of NETs is an important step in the immune response, and even though NETs are beneficial in terms of pathogen containment, they have been associated with severe lung damage and can worsen the disease course [25, 26]. Both excessive NET formation or its impaired degradation have pathogenic implications [7, 20, 27].

Continuous influx of inflammatory cells and mediators to the site of infection leads to tissue deterioration and formation of tuberculous granulomas, organized spherical structures composed of various cell types [28, 29]. Neutrophils in specific mediate the MTB containment through the production of granulomas [30]. The classic TB-granuloma contains a caseous (necrotic) core, surrounded by epithelioid macrophages, and an outer cuff of lymphocytes and macrophages [28], neutrophils, monocytes, dendritic cells, and T cells [1, 13]. Among other changes, the presence of caseating granulomas is a hallmark of TB [31, 32]. However, there is considerable variability in granuloma types even within the same host. As such, non-caseating granulomas often appear as well [28]. Even though granulomas have initially been described in the containment of MTB growth [1, 13], recent studies questioned their protective role [1, 33]. MTB can reside in granulomas for decades and progress to active TB at any point. In an immunocompromised state, liquefaction of granuloma caseum may lead to bacterial replication. Fusion of granulomas may lead to cavity formation and intrapulmonary spread of MTB [30]. Furthermore, there are other types of diseases with granulomas and sarcoidosis (SARC) is mostly associated to TB.

SARC is characterized by the formation of granulomas, most commonly in the lungs, lymph nodes, skin and eyes [34]. Some individuals may have no symptoms, while others experience fatigue, cough, skin rashes, joint pain, and enlarged lymph nodes [35]. The clinical picture depends on the stage and prevalence of SARC. Although SARC and TB can have similar features, they are distinct diseases with different causes, pathophysiology, and treatments. Whereas TB is caused by MTB, the exact cause of SARC remains unknown. Conflicting reports on MTB as a putative cause of SARC are available [36]. Mycobacterial antigens may contribute to SARC development in some cases [34, 37]. Whereas TB is primarily characterized by caseating granulomas containing necrotic tissue indicating bacterial infection [38], non-caseating granulomas without central necrosis have been considered a hallmark of SARC [31, 34]. However, there is evidence that caseating granulomas also exist in SARC [39].

Apical periodontitis (AP), most commonly caused by oral bacteria infecting the tooth pulp, is yet another condition characterized by granuloma formation [40]. It is categorized into acute and chronic inflammation [41]. Substantial number of inflammatory cells, primarily neutrophils, are recruited following infection. Granuloma incidence in AP ranges from 46 to 94% [42]. In patients with persistent AP, endodontic microsurgery may result in a predictable treatment outcome with persistent peri-radicular lesions [43].

In this study, we assessed circulating levels of NET degradation products in patients with distinct types of TB. We report increased circulatory NET formation in TB patients in relapse, and TB patients with extensive tissue damage, thus highlighting the importance of balancing NET formation to prevent tissue destruction and analyzing circulating NET parameters to monitor the risk of disease relapse. We further examined tissue NET formation in caseating granulomas in pulmonary and extrapulmonary TB by employing immunofluorescence. We investigated tissue NET formation in other diseases with granulomas, including SARC and AP. Tissue NET formation occurs in both pulmonary and extrapulmonary TB, and appears as a common feature of caseating granulomas. Furthermore, DNase-resistant Z-form DNA accumulates in the granulomas, and is most abundant in the non-caseating type. Given the association of TB with hypercoagulation and consequently thromboembolism, we further investigated pulmonary vessels for occlusions. We detected extensive pulmonary vascular occlusions in TB; these vessels harbored neutrophil aggregates.

## Materials and methods

### Patients

This research lasted from January 01, 2021 to December 31, 2021 at Kharkiv Anti-TB Dispensary #1, a regional capital city with approximately 1.4 million inhabitants in eastern Ukraine. According to the World Health Organization (WHO), the TB incidence in Ukraine was 90 per 100,000 inhabitants in 2022 (provided at https://worldhealthorg.shinyapps.io/tb_profiles/?_inputs_&entity_type = %22country%22&iso2 = %22UA%22&lan = %22EN%22). In line with Ukrainian national TB guidelines, all persons with presumptive TB should consult a TB-doctor specialized in TB diagnosis and treatment. For diagnostic purposes, a series of microbiological tests, chest radiography and basic blood tests were performed by the phthisiatrician. Once diagnosed, TB patients usually start treatment in the outpatient departments in case of limited disease; in case of extensive disease, they are admitted to a TB hospital until sputum conversion is achieved. The study population consisted of 91 patients in total (69 male, 22 female). All patients were sputum positive with the Xpert MTB/RIF [44], culture and drug-susceptible test for 1st and 2nd line anti-TB drugs. The patients enrolled for this study were divided into four groups. Group 1 included the patients with susceptible TB, group 2 was composed of the patients with monoresistant TB, while group 3 was composed of the patients with MDR/Pre-XDR/XDR-TB. Group 4 comprised the conditionally healthy individuals. Detailed characteristics of the study population are shown in Table 1. All treatment regimens were adopted from standardized WHO guidelines and national protocol of Ukraine for the diagnosis and treatment of tuberculosis as follows: Group 1 patients received isoniazid, rifampicin, pyrazinamide, and ethambutol for the duration of 2 months, followed by isoniazid and rifampicin for the duration of 4 months. Group 2 patients were given either rifampicin, pyrazinamide, ethambutol and levofloxacin (isoniazid-resistant), or the same treatment as group 1 with the addition of a second-line drug instead of a resistant drug (ethambutol- or pyrazinamide-resistant) or second-line drugs (rifampicin-resistant) for the duration of 6 months. Group 3 patients were put on a standard regimen of second-line drugs (bedaquiline, linezolid, levofloxacin, clofazimine, cycloserine or delamanid), considering a mutation in *rpoB* resistance gene of MTB was identified employing GeneXpert. Once the results of bacteriological examinations were obtained, the treatment regimen was adapted to the drug susceptibility of MTB. The treatment lasted 9 months for MDR TB, and 18-20 months for Pre-XDR/XDR-TB.

**Table 1:** Overview of clinical parameters of TB patients (*n* = 91) used for correlation analyses^a^.

Clinical parameters (TB patients)	Gender	
	Male (*n* = 69)	Female (*n* = 22)
Age (years)	47 (range: 28–72)	49 (range: 30–69)
Smoking	44 (64%)	10 (45%)
Radiologic evidence of tissue destruction	46 (67%)	15 (68%)
New case	48 (70%)	17 (77%)
Relapse	19 (28%)	5 (23%)
Failure of treatment and loss to follow-up	3 (2%)	0 (0%)
Susceptible TB	30 (43%)	6 (27%)
Monoresistant TB	3 (2%)	5 (23%)
Multi-drug-resistant (MDR) TB	25 (36%)	8 (36%)
Pre-extensively-drug-resistant (Pre-XDR) TB	9 (13%)	2 (9%)
Extensively drug-resistant (XDR) TB	3 (4%)	1 (5%)
**Leukogram analyses**	**Normal neutrophil count (40–60% total)**	**Neutrophilia (** **>** **70% total)**
All patients	60/91 (65.9%)	31/91 (34.1%)
Patients in relapse	14/60 (23.3%)	10/31 (32.3%)
Patients with tissue destruction	38/60 (63.3%)	23/31 (74.2%)

^a^Data are given as mean values (with range, where applicable) unless stated otherwise. Parentheses contain the percentage of the population with the corresponding clinical parameters.

Lung biopsies of patients with TB (*n* = 4) were obtained from Kharkiv, Ukraine. Lymph node biopsies of patients with TB (*n* = 3) and samples from 35 patients with AP were obtained from Trier, Germany. Lung (*n* = 6) and lymph node (*n* = 6) biopsies of patients with SARC were provided by the tissue bank of the Comprehensive Cancer Center (CCC, Erlangen, Germany). All samples arrived embedded in paraffin, and were further used for histological examination as described in more detail in “Immunohistochemistry (IHC) and immunofluorescence (IF)”.

#### Randomization and exclusion/inclusion criteria

Inclusion criteria in this study were the presence of MTB culture-confirmed pulmonary TB, whereas exclusion criteria were the following: HIV/AIDS, hepatitis A, B, and C, pregnancy; lactation; the inability to give written consent to participate in the study; severe psychosis; poliomyelitis (including in medical history); acute hepatic and/or renal insufficiency; amyloidosis and malignant oncological pathology. Randomization was performed on the basis of PC-generated successive random numbers. Ten patients were excluded from the study as a result of the exclusion criteria as early losses. One patient refused to participate in the study after randomization.

#### Microbiological examinations

Standard microbiological examinations were performed at admission, after 2 and 6 months following treatment initiation using smear microscopy (Ziehl-Neelsen staining), mycobacterial culture on solid (Lowenstein–Jensen) and liquid ВАСТЕС Mycobacterial Growth Indicator Tube (MGIT-960) medium. Automated detection of MTB using Xpert MTB/RIF (Cepheid, Sunnyvale, CA, USA) testing was carried out at admission using sputum samples when available. Phenotypic drug susceptibility testing (DST) of MTB was performed at admission using solid and/or liquid media when available. The spectrum of tested drugs using DST included isoniazid, rifampicin, ofloxacin, levofloxacin, moxifloxacin, cycloserine, pyrazinamide, ethambutol, streptomycin, ethionamide, amikacin, capreomycin, kanamycin, para-amino-salicylic acid, bedaquiline and linezolid.

#### X-ray examination

Pathological features (e.g., the disease severity and changes in the localization of the process in lungs) were assessed via X-ray examination. We observed various clinical and radiologic forms of pulmonary TB.

#### Blood count and biochemical tests

Blood samples were collected from the patients to count leukocytes and their types via leukogram analysis, as well as to evaluate the erythrocyte sedimentation rate (ESR). Sampling was performed the next day after patient admission to the hospital, before starting anti-TB treatment, between 8:00 and 9:00 a.m. on an empty stomach. At this point, the group allocation was unknown. Note that the use of anti-TB drugs was after taking blood samples. Total leukocyte counts were in the range of 1.4–19.4 × 10^9^/L (mean 7.4 × 10^9^/L), whereas neutrophil counts were in the range of 0.9–15.1 × 10^9^/L (mean 5.1 × 10^9^/L), thus attributing to 39.0–96.2% of the total leukocyte count (mean 66.2%). Note the prevalence of neutrophilia in 1/3 of the patients, whereas the other 2/3 had normal neutrophil counts. The samples were further used to prepare blood serum in which the activity of alanine aminotransferase (ALT) and aspartate aminotransferase (AST), the levels of total bilirubin and total protein were determined in a certified clinical laboratory.

### Assessment of neutrophil activation and NET formation in blood serum

Blood samples from normal healthy donors (NHD) and TB patients were collected in Kharkiv, Ukraine. The serum was obtained by centrifugation, stored at −70 °C, and further processed in Erlangen, Germany. NET markers, including cell-free DNA (cfDNA), neutrophil elastase (NE)-DNA and myeloperoxidase (MPO)-DNA complexes, and NE activity, were further investigated as described below in more detail. The investigators were blinded to the group allocation during the experiment and when assessing the outcome.

#### Detection and quantification of cell-free DNA

The amount of cfDNA in the sera was determined by employing the Quant-iT™PicoGreen™dsDNA Assay-Kit (P11496, Thermo Fisher Scientific Inc., Waltham, MA, USA) according to manufacturer’s instructions. Samples were prepared as briefly described elsewhere [45], and the fluorescence (Ex.: 485 nm, Em.: 535 nm) was measured with the Infinite F200 PRO fluorescence plate reader (Tecan, Männedorf, Switzerland). Final concentrations of cfDNA were calculated using the supplied DNA standard.

#### Enzyme-linked immunosorbent assay (ELISA) of MPO–DNA & NE-DNA complexes

The presence of MPO–DNA and NE-DNA complexes in the serum was determined using the modified ELISA, as already described in [45]. Briefly, Nunc MaxiSorp™ 96-well plates (#442404, Thermo Fisher Scientific Inc., Waltham, MA, USA), pre-coated with 100 µL/well of either rabbit anti-human MPO antibody (07-496-I, Merck KGaA, Darmstadt, Germany, 1:2000) or rabbit anti-human NE antibody (MAB91673, R&D Systems, Minneapolis, MN, USA, 1:1000) were washed thrice with a wash buffer (0.05% Tween-20 in Dulbecco´s Phosphate Buffered Saline (DPBS, Thermo Fisher Scientific Inc., Waltham, MA, USA) and blocked with 3% BSA (SC-2323, Santa Cruz Biotechnology Inc., Dallas, TX, USA) in Dulbecco´s Phosphate Buffered Saline (DPBS, Thermo Fisher Scientific Inc., Waltham, MA, USA) for 2 h at RT, shaking. The washing step was repeated, and serum added to each well and incubated for 2 h at RT, shaking. Another washing step followed, and the anti-DNA POD from Cell Death Detection Kit (11544675001, Merck KGaA, Darmstadt, Germany), diluted 1:40, was added for 90 min at RT in the dark with shaking. After one last washing step, the reaction was developed by incubating the TMB Substrate (#421101, BioLegend, San Diego, CA, USA) for 30 min at RT. The reaction was stopped by the addition of 25% of H_2_SO_4_ (#122448.1211, AppliChem GmbH, Darmstadt, Germany). Finally, the absorbance was read at 450 nm with a reference at 620 nm, using a Sunrise microplate reader (Tecan Group Ltd.).

#### Measurement of NE activity

To assess the activity of NE in the sera of patients with TB, 10 µL of serum and 100 µM fluorogenic NE substrate (MeOSuc-AAPV-AMC, sc-201163, Santa Cruz Biotechnology Inc., Dallas, TX, USA) per well were diluted in Dulbecco´s phosphate buffered saline (DPBS, Thermo Fisher Scientific Inc., Waltham, MA, USA) in a 384-well plate (#3680, Corning Inc., Corning, NY, USA). The conversion of the fluorogenic substrate (Ex.: 360 nm, Em.: 465 nm) by NE in the serum was assessed by an increase in the mean fluorescence intensity (MFI) using the Infinite F200 PRO fluorescence plate reader (Tecan, Männedorf, Switzerland) for 20 h at 37 °C. The first value was subtracted as the baseline from the endpoint measurement taken after 3.5 h.

### Immunohistochemistry (IHC) and immunofluorescence (IF)

Paraffin-embedded tissue sections were used for immunohistochemistry analyses. Hematoxylin and eosin (HE) and CD31 staining were performed using standardized staining procedures. Immunofluorescence staining for neutrophil-associated markers: NE, MPO, and citrullinated histone H3 (citH3), as well as different isoforms of DNA (B-form and Z-form), followed. Due to a limited number of sections, staining for B-form and Z-form DNA was not conducted on TB lymph node biopsies. The blocking was performed with a blocking buffer containing 10% FCS and 2% BSA in DPBS. For this purpose, we used the following antigen-specific primary antibodies: antibody against NE (Abcam, ab68672, 1:100), MPO (Abcam, ab9535, 1:200), citrullinated histone H3 (citH3, Abcam, ab5103, 1:300), B-DNA (Abcam, ab27156, 1:50), and Z-DNA (Absolute Antibodies, Ab00783-23.0, 1:100). Thereafter, we used the following secondary antibodies conjugated with fluorophores: goat anti-rabbit Cy5 (Jackson ImmunoResearch, 111-175-144, 1:400) for NE, MPO, citH3 and Z-form DNA, and goat anti-mouse PE (Jackson ImmunoResearch, 115-116-071, 1:400) for B-form DNA. Hoechst33342 (Molecular Probes, 1:5000) or 4′,6-diamidin-2-phenylindol (DAPI, Thermo Fisher Scientific Inc., Waltham, MA, USA, 0.2 μg/mL) were employed as general DNA dyes. Tissue sections were mounted with DAKO fluorescence mounting medium (Agilent Technologies) and visualized by fluorescence microscopy (Aperio Versa 8, Leica Biosystems).

### Statistical evaluation

Graphs and figures were created using GraphPad Prism Version 9.0.2 (161) (GraphPad Software, LLC, San Diego, CA, USA) and Microsoft Office Professional Plus 2019 (Microsoft, Redmond, WA, USA). For statistical analyses, Mann–Whitney test, Kruskal–Wallis test, ordinary one-way ANOVA, or two-way ANOVA were employed, as indicated in the figure legends. To assess the distribution of the data and evaluate normality assumptions where necessary, normality tests were conducted. Statistical correlations were calculated using IBM SPSS Statistics Version 28.0.0.0 (190) (IBM, Armonk, NY, USA). Kolmogorov–Smirnov test was used for the assessment of normality. The parametric ANOVA test with post hoc analysis was used to statistically process the obtained numerical data. The difference was considered to be statistically significant at *P* < 0.05. All data are presented as mean ± standard deviation (s.d.), as also indicated in the figure legends. The study was prospective and cohort, with respective inclusion and exclusion groups. Available patients were included, resulting in statistical power below 0.8.

### Ethical considerations

This study was carried out in accordance with the Declaration of Helsinki. It was approved by the Ethics Committee of the Kharkiv National Medical University, Kharkiv, Ukraine (minutes No 1 dated February 5, 2021). Informed consent was obtained from all subjects involved in the study.

## Results

### Circulating NET degradation products are increased in TB patients, characteristic to patients in relapse and associated with lung destruction

**Fig. 1:**
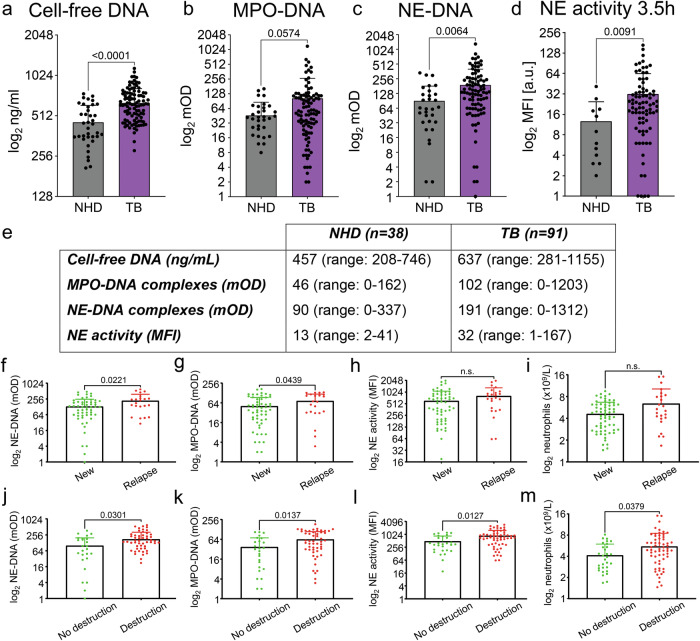
Circulating NET degradation products are associated with lung destruction and relapse in TB patients. We detected NETs by the quantification of circulating NET degradation products and found that they are elevated in patients with TB when compared to NHD. **a** We quantified cfDNA in the sera by PicoGreen fluorescence, **b** MPO–DNA and **c** NE-DNA complexes by ELISA and **d** NE activity by the conversion of a specific fluorogenic substrate. **e** Summarizes mean values and ranges for all parameters. **f**–**m** Clinical parameters were associated with the results obtained by NET formation analyses of TB sera. **f** NE-DNA complexes, **g** MPO–DNA complexes, **h** NE activity and **i** neutrophil counts in the sera of TB patients, associated with the type of TB case: new (green) versus relapse (red). Note that NET formation is increased in the sera of TB patients in relapse. **j** NE-DNA complexes, **k** MPO–DNA complexes, **l** NE activity and **m** neutrophil counts in the sera of TB patients, grouped by the extent of tissue destruction: no destruction (green) or destruction (red). Note that NET formation, NE activity and neutrophil counts are increased in patients with tissue destruction. All statistical analyses were performed employing the Mann–Whitney test. Data are presented as mean ± standard deviation (s.d.). At least three technical replicates were obtained for each data set. NHD normal healthy donors, TB tuberculosis, MPO myeloperoxidase, NE neutrophil elastase, MFI mean fluorescence intensity, n.s. not significant.

To compare circulatory NET degradation products, we analyzed the sera for cfDNA and complexes of DNA with MPO or NE (Fig. 1a–c, e). cfDNA and MPO–DNA or NE-DNA complexes were elevated in the sera of patients with TB, when compared to NHD. The difference for cfDNA and NE-DNA complexes reached statistical significance. Next, we assessed NE (Fig. 1d, e) and detected increased proteolytic activity in the sera of patients with TB, when compared with NHD.

When we grouped the results of NET formation analyses to clinical parameters summarized above in Table 1, we observed significant differences in NET formation for the type of TB case (new case versus relapse) and tissue destruction (no destruction versus destruction). In patients in relapse, NE-DNA complexes and MPO–DNA complexes were increased (Fig. 1f, g), when compared to new cases. The same was observed for patients who had tissue destruction, compared to those without (Fig. 1j, k). In the sera of patients in relapse, we observed a trend towards increased NE activity (Fig. 1h) and higher neutrophil counts (Fig. 1i), whereas in patients with tissue destruction, NE activity (Fig. 1l) and neutrophil counts (Fig. 1m) were significantly increased.

Using IBM SPSS Statistics Version 28.0.0.0 (190) (IBM, Armonk, NY, USA), we further correlated the results of NETs assays to clinical parameters, and discovered two statistically significant correlations: [1] tissue destruction with NE-DNA complexes (*P* = 0.009) and [2] antibiotic resistance, as determined by the Xpert MTB/RIF test, for multi-drug-resistant (MDR) type of TB and MPO–DNA complexes (*P* = 0.041).

Further analysis of NET markers in terms of TB resistivity revealed NETs in all types of resistant TB (Supplementary Fig. S1), albeit showing no significant differences in NET formation, NE activity or neutrophil counts between the various TB subtypes. However, these findings are limited by a relatively small cohort size for monoresistant (*n* = 7), Pre-XDR (*n* = 11) and XDR (*n* = 4) types of TB. An increase in the number of patients would perhaps show a significant relation(s) between NET formation and different type(s) of resistant TB. Finally, simple linear regression of NET formation to neutrophil counts (Supplementary Fig. S2) revealed no significant correlations between cfDNA, NE-DNA or MPO–DNA complexes and neutrophil counts in TB patients (Supplementary Fig. S2a), also within the cohorts of interest (Supplementary Fig. S2b, c). Interestingly, with neutrophilia, a higher percentage of patients experience relapse or extensive tissue destruction (Table 1).

### NET formation is a hallmark of caseating granulomas in TB and SARC

As (I) NETs assays revealed increased NET formation in TB sera, and (II) NETs and NE activity were increased in patients with tissue destruction, we analyzed lung and lymph node biopsies from TB patients using IF for tissue-resident NETs. We stained for the NET marker proteins NE and MPO as well as citrullinated chromatin (citH3) and extended the analysis to lung and lymph node biopsies from SARC patients. Supplementary Table S1 displays an overview of all TB and SARC samples, sorted according to their abundance upon visual estimation. We detected strong signals for NE and MPO in biopsies of lymph nodes and lungs of patients with TB and SARC, respectively; the latter displayed the maximum MFI in the granulomas. NETs showed co-localization of the widely spread chromatin with these marker proteins, whereas clear segregation of nuclear chromatin and cytoplasmic granules was observed in neutrophils. Furthermore, citH3 was highly expressed in lymph node granulomas of both TB and SARC. As NET markers were predominantly abundant in the granulomatous regions of our biopsies, we further focused on the granulomas grouping them into caseating and non-caseating subtypes by the histopathological evaluation of hematoxylin and eosin (HE)-stained tissue sections [46, 47].

**Fig. 2:**
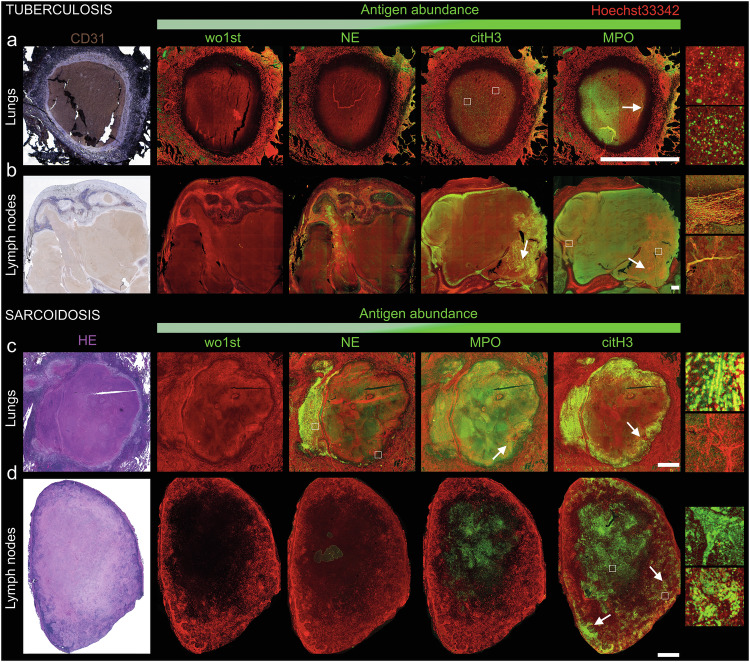
Caseating granulomas in TB and SARC contain NETs. Various staining for NETs (NE, citH3, MPO) in **a**, **b** TB and **c**, **d** SARC. Upper panel: **a** lung granuloma and **b** lymph node granuloma from a representative TB patient. Lower panel: **c** lung granuloma and **d** lymph node granuloma from a representative SARC patient. All antigens (NE, citH3, MPO) are shown in green, and sorted according to their abundance from left to right (low to high). Note the difference between TB and SARC; MPO is most abundant in TB, citH3 in SARC. Mock staining without the primary antibody (wo1st) served as control. Hoechst33342 was used as a DNA dye (red). Arrows mark NET-rich regions; white squares mark NETs which have been further enlarged and are displayed to the right of the original images. We applied additional tonal corrections to the insets to improve visibility. Labeled and mock-stained images from the same tissue sample were co-processed. HE staining, purple; CD31 staining, brown. Bars represent 1 mm.

Interestingly, caseating granulomas were present not only in TB (Fig. 2a, b) but also in lung (67%) and lymph node (17%) biopsies from SARC patients (Fig. 2c, d). TB granulomas further stained positive for CD31, a molecule involved in leukocyte adhesion and transendothelial migration. Importantly, we detected a strong, widely spread signal for NET markers in all caseating granulomas (i.e., in the TB lymph nodes and the SARC lungs). A lower signal was detected in the caseating granulomas in TB lungs and SARC lymph nodes. DNA was counterstained employing Hoechst33342. To confirm NET formation, we analyzed all images for the co-localization of NET markers with DNA and observed NETs in all caseating granulomas. Importantly, non-caseating granulomas, detected mostly in the lymph nodes of SARC patients, showed no signs of NET formation (Supplementary Fig. S3). This was indicated by low signals of extracellular DNA, and segregation of NET markers in the cytoplasm of viable neutrophils. Nevertheless, we detected neutrophil degranulation and histone citrullination in non-caseating granulomas. We further detected no NETs in patients with apical periodontitis within the granulomatous regions that lacked necrosis (Supplementary Fig. S4).

### Granulomatous regions are enriched in DNase-resistant Z-form DNA

**Fig. 3:**
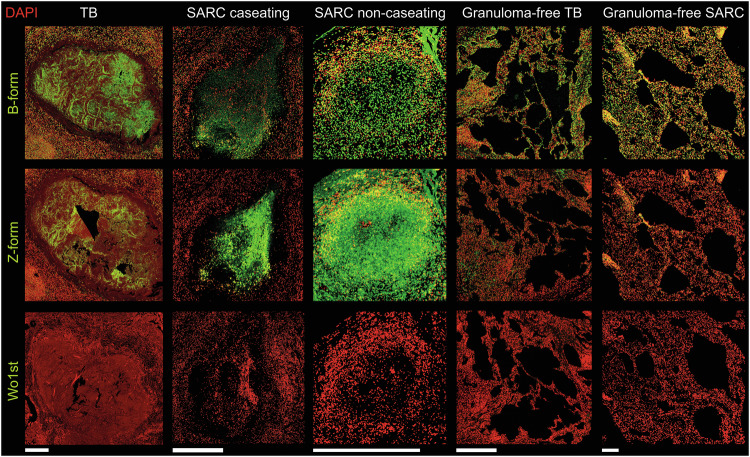
Granulomas harbor the DNase-resistant Z-form DNA. Detection of B and Z isoforms of DNA by IF in caseating granulomas in TB and caseating or non-caseating granulomas in SARC, as well as in parts without granuloma. Antibodies recognizing B- or Z-form DNA and the low molecular weight DNA stain DAPI are displayed in green and red, respectively; Mock staining without a primary antibody served as control. TB tuberculosis, SARC sarcoidosis. Bars represent 500 µm.

We further stained the caseating granulomas in TB and caseating as well as non-caseating granulomas in SARC for two distinct isoforms of DNA: B-form and Z-form. Whereas all granulomas and non-granulomatous tissue contained the DNase-sensitive B-form DNA; granulomas further contained the DNase-resistant Z-form DNA (Fig. 3), while non-granulomatous tissue contained considerably less Z-form DNA.

**Fig. 4:**
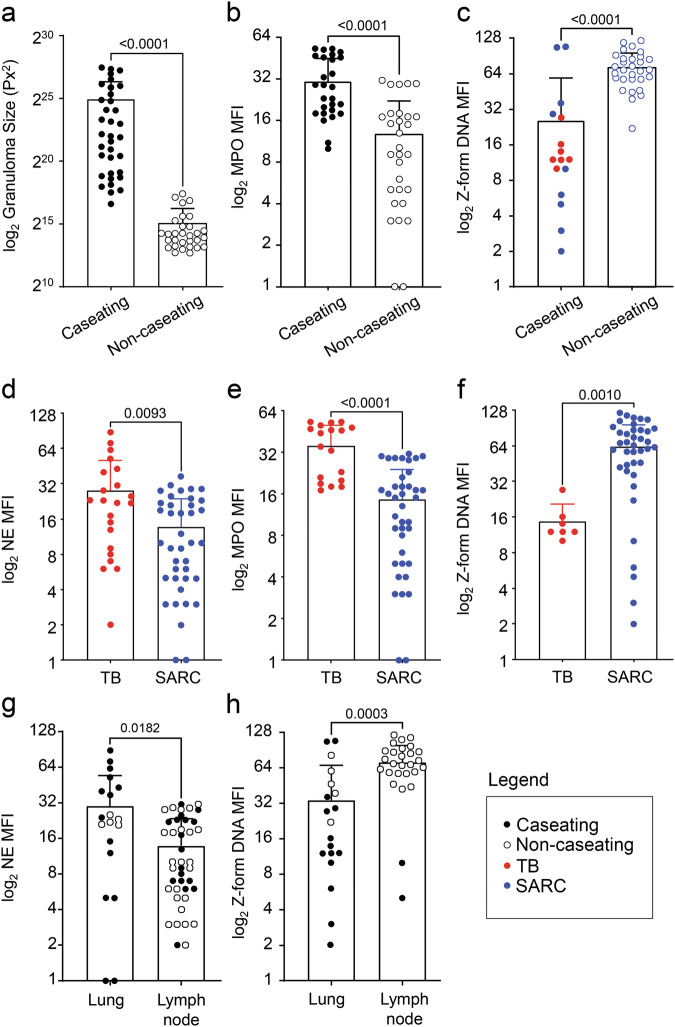
Morphometry of various granulomas. We performed morphometry analyses of all granulomas, and grouped them based on caseation, disease, and type of tissue. **a**–**c** Caseating versus non-caseating granulomas; **d**–**f** TB versus SARC; and **g**, **h** lungs versus lymph nodes. Note the further discrimination of TB and SARC in (**c**), and of caseating versus non-caseating granulomas in (**g**, **h**). Note that caseating granulomas are larger than the non-caseating ones and that NET markers and Z-form DNA are more abundant in TB and SARC granulomas, respectively. All statistical analyses were performed employing the Mann–Whitney test. Data are presented as mean ± standard deviation (s.d.).

In-depth analysis of all granulomas by morphometry further revealed differences in the size of granuloma (Fig. 4a); with caseating granulomas being larger than the non-caseating ones. Moreover, caseating granulomas were enriched in MPO (Fig. 4b), and non-caseating granulomas in Z-form DNA, respectively (Fig. 4c). TB granulomas were more abundant in NE and MPO (Fig. 4d, e), and SARC granulomas in Z-form DNA (Fig. 4f). We also detected differences with respect to the type of tissue studied, as lung granulomas were more abundant in NE (Fig. 4g), and lymph node granulomas in Z-form DNA (Fig. 4h).

**Fig. 5:**
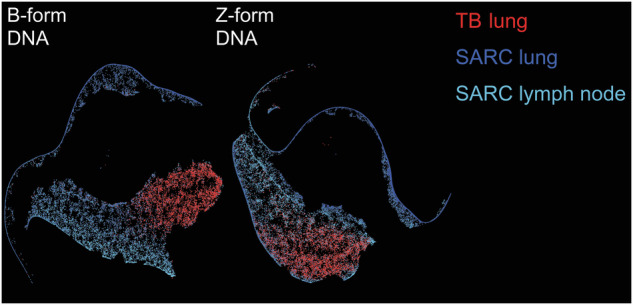
TB and SARC granulomas differ in B-form or Z-form DNA staining. FIt-SNE plots obtained from the morphometric analysis of B-form DNA or Z-form DNA MFI and grouped according to the type of disease and the affected organ. For further differentiation of caseating and non-caseating granulomas see Supplementary Figs. S4 and 6. TB tuberculosis, SARC sarcoidosis.

Next, we performed morphometry by automatic cluster assignment employing FIt-SNE. Granulomas were heterogenous in staining for NET markers (Supplementary Fig. S5). The signal intensity for B- and Z-form DNA differs between TB and SARC; TB forms a cluster apart from SARC (Fig. 5).

**Fig. 6:**
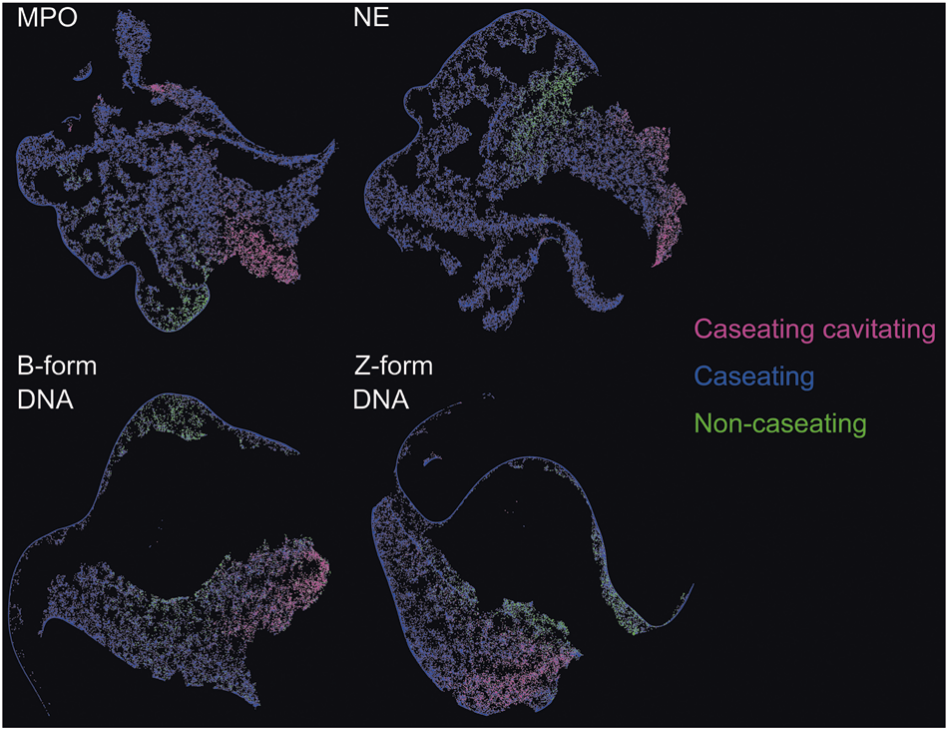
Caseating and non-caseating granulomas can be distinguished by staining for NET markers and DNA isoforms (B/Z). FIt-SNE plots are calculated by morphometry of MPO, NE, B-form DNA, or Z-form DNA MFI values, grouped according to the subtypes of granuloma; caseating (cavitating) versus non-caseating.

Caseating granulomas can further be differentiated from the non-caseating ones in staining for both NET markers and isoforms of DNA (Fig. 6).

### In patients with TB the pulmonary vessels are frequently occluded by neutrophil aggregates

**Fig. 7:**
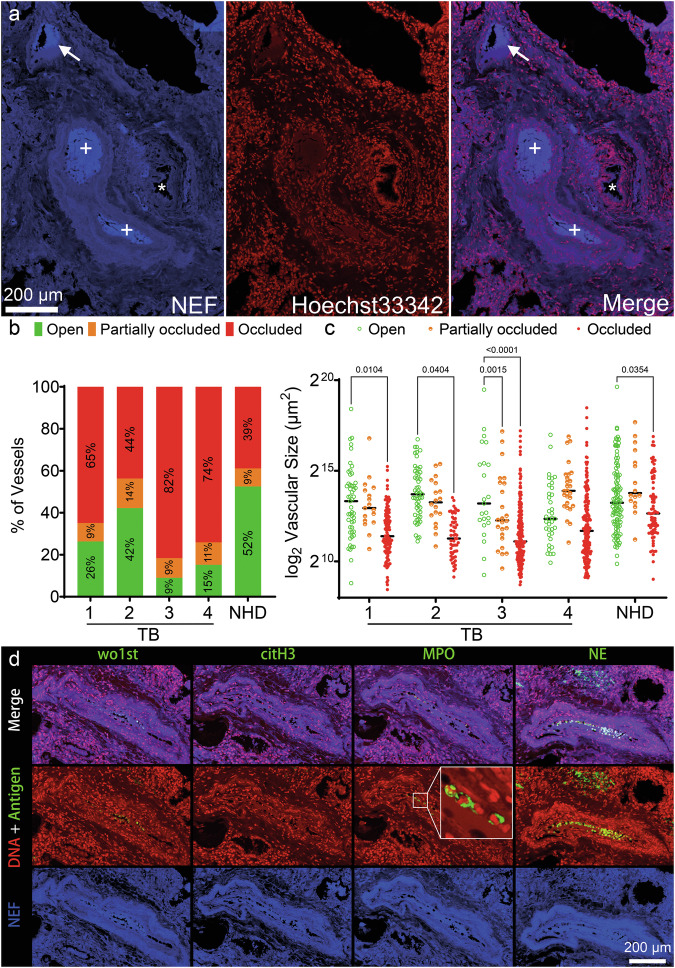
Most pulmonary vessels in the granulomatous regions of TB are occluded by seemingly viable neutrophils. Native endogenous fluorescence (NEF) was used to detect pulmonary vascular occlusions in TB biopsies (*n* = 4). **a** One of the analyzed lung sections showing an open vessel (*), a partially occluded vessel (arrow), and occluded vessels (+). **b** The percentage of pulmonary vessels which are open (green), partially occluded (orange), or completely occluded (red). *P* = 0.0065 for partially occluded versus occluded. **c** The size of open, partially occluded, and occluded vessels (single dots represent individual blood vessels). **d** Staining for NET-associated markers (citH3, MPO, NE) in occlusions. Note that citH3 is absent and NE or MPO showed cytoplasmic appearances, typical for viable neutrophils. White square marks neutrophil aggregates which have been further enlarged to improve visibility. Statistical analyses were performed using the two-way ANOVA. NHD normal healthy donor.

As TB has been associated with hypercoagulation and thromboembolism [48], we further analyzed lung biopsies in TB for the presence of vascular occlusions (Fig. 7a) using a label-free method which employs native endogenous fluorescence (NEF) [49]. We differentiated between open, partially occluded, and occluded vessels, and detected all types (Fig. 7b). Importantly, the majority of the vessels in TB were occluded; these vessels were usually the smallest (Fig. 7c). Given that previous studies linked vascular occlusions to NETs in the context of COVID-19 [50, 51], we further analyzed pulmonary vascular occlusions in TB for NET-associated markers. Within these occlusions, we detected no citH3-positive material; rather neutrophil aggregates expressing cytoplasmic proteins (NE, MPO). These aggregates retained the morphology of intact neutrophils, as indicated by the lack of co-localization of nuclear chromatin with cytoplasmic proteins, otherwise characteristic to NETs (Fig. 7d).

## Discussion

There is a growing demand for diagnostic tests capable of identifying individuals with active TB, or latent TB at the risk of progressing to active TB [52]. Plasma levels of NE and MPO are elevated in individuals with active TB when compared to latent TB, and tend to normalize with treatment. Systemic levels of MPO show potential to differentiate between active and latent TB [53]. Moreover, plasma NET levels correlate with disease severity, and their quantity decreases after therapy [54]. It is unclear whether this increase is initially beneficial, due to the early pro-inflammatory nature of NETs, and in time detrimental, as a consequence of excessive release, inefficient clearance, or both [26]. In this study, we detected increased NET formation in TB sera, when compared to physiological low-level NET formation in the circulation of NHD, as reported earlier [11, 54]. In addition, we detected an increased enzymatic activity of circulating elastase. Despite significant research in this area, no connection between serum NETs levels and different types of TB has been made so far. By employing NE-DNA and MPO–DNA complex ELISA, we report an abundance of NETs in the sera of TB patients in relapse compared to new cases. These findings suggest that NETs contribute to, or may even trigger, patient relapse. Therefore, circulating NE- or MPO–DNA complexes can be used as a reliable risk marker for relapse of TB; the ELISA is suitable for high-throughput processing. We report increased circulatory NET formation and NE enzymatic activity in patients suffering from extensive tissue destruction. This highlights the detrimental role of NETs in TB-associated tissue damage [25]. The inhibition of NE activity, and/or NET formation or the augmentation of NET clearance seem to be promising approaches to mitigate tissue damage and potentially reduce the risk of disease relapses. Considering that NET formation in TB did not correlate with neutrophil counts, it could be a reflection of the cell activation state rather than cell quantity. Interestingly, a higher percentage of patients experienced relapse or extensive tissue destruction if in the neutrophilic group, neutrophils may also contribute to patient relapse or tissue destruction by other mechanisms than NET formation.

As we observed increased NET formation and NE activity in the circulation of TB patients with tissue destruction, we investigated the lungs and lymph nodes of TB and SARC patients for the presence of neutrophils and NETs. Considering a substantial degree of heterogeneity between various types of granulomas, the outcomes in individual granulomas have already been connected with the TB spectrum of risk [28]. Despite many similarities between TB and SARC, only a limited number of studies have directly compared NET formation in patients with SARC (mainly non-caseating) and TB (mostly caseating). In accordance with previous reports on murine lung lesions and areas of caseous necrosis [55], we detected NETs in human pulmonary TB samples. So far, NETs have been described in caseating granulomas in TB, but not in non-caseating granulomas in SARC [56]. Here we report NETs in granulomas in both TB (frequently caseating) and SARC (mainly non-caseating) patients. Currently, no evidence of NET formation in extrapulmonary SARC is available. Thus, we analyzed the affected lymph nodes and observed that NET formation is not restricted to pulmonary tissues. Instead, we detected NETs also in other caseating granulomas. It was not surprising that we detected no NET formation in the non-caseating granulomas, as areas with caseation have been associated with an increased bacterial load, compared to non-caseating early lesions [57]. This might affect the neutrophil activation status.

Under physiological conditions, B-form DNA is the most abundant DNA isoform. Z-form DNA is less abundant due to its high intrinsic energy. B- and Z-form DNA also differ in their susceptibility to degradation by DNase; Z-form DNA reportedly resists DNaseI [58, 59]. Whereas non-granulomatous regions in TB and SARC contain predominantly the B-form DNA, the granulomas also contain the DNase-resistant Z-form DNA. The Z-form may indicate mature bacterial biofilm formation [60]. However, we favor another explanation for the B-DNA to Z-DNA conversion (B-Z-Flip). Mammalian genomes harbor d(G − T)_n_:d(C − A)_n_ stretches that can perform B-Z-Flip under reasonable levels of negative superhelical stress. In fact, sequences as short as d(GT)_6_:d(AC)_6_ can adopt the Z-conformation. The repetitive Alu sequences contain huge amounts of potentially Z-forming sequences and make up 10% of the human genome. Chromatin processing enzymes unwind DNA in their wake and thus promote the B-Z-Flip. Once nucleated, it tends to proceed cooperatively [61]. During NET formation citrullination of DNA-bound histones reduces their positive charges, weakens their binding to DNA, and induces citrullination-dependent histone release. This leads to chromatin unfolding and decondensation [62], and is associated with local negative superhelical stress and is accompanied by the formation of DNA patches with Z-conformation; the latter to be immune detected by Z-form DNA specific antibodies [63]. As Z-form DNA resists degradation by DNaseI [64], the action of this enzyme preferentially degrades the B-form and preserves the Z-form DNA, which consequently accumulates in the tissue.

This is an important insight, would the application of DNases be considered a potential therapeutic strategy for targeting excessive NET formation. The use of recombinant human DNaseI (dornase alfa, Pulmozyme, Roche) has already been shown efficient in treating other pulmonary diseases, such as asthma, COVID-19 and others [65].

Interestingly, we detected Z-form DNA in both caseating and non-caseating granulomas, despite the lack of exuberant cell death in the latter. However, both types of granulomas exhibited extensive tissue citrullination, indicative of cellular stress and inflammation [66]. Citrullination precedes cell death and does not occur completely parallel to it. Furthermore, during vital NET formation neutrophils release NETs but remain viable and maintain their cellular functions [67].

Complete mechanisms by which MTB evades NETs have not yet been fully elucidated. For example, bacterial species from *Streptococcus* and *Staphylococcus* genera produce DNases which impair NET formation [7]. On the other hand, *Vibrio cholerae* first induces NET formation, followed by secretion of two different extracellular nucleases to degrade the DNA component of NETs [68]. The degradation of NETs serves as a defense mechanism, and extracellular DNA is utilized as a nutrient source. MTB, found in TB and debatably also in some SARC patients [31], is an important source of extracellular nucleases, such as Rv1108c, Rv2090, Rv0631c, Rv3674c, Rv0629c/Rc0630c, and Rv0888 [69]. Interestingly, MTB-derived nuclease Rv0888 has also been shown to induce NET formation [70] and to degrade DNA as well as RNA [71]. Whether Rv0888 is also capable of degrading the DNA of NETs, especially the Z-form DNA-rich areas, remains elusive. The impact of these nucleases on NETs presents itself as an interesting direction of research for further studies.

Prior research has highlighted differences in the vascular structure within TB granulomas as compared to healthy lung tissue [72]. Our study further reveals a significant degree of vascular occlusions in MTB-infected lungs, particularly affecting the smaller blood vessels. As MTB takes advantage of the blood vessels to disseminate [6], it is not surprising that we detected neutrophils in vascular occlusions of pulmonary TB, as neutrophils likely migrate to this place to combat the pathogen.

NET formation is an essential part of the immune response against a plethora of diseases. It constrains infections, presumably by restricting bacterial spread. However, the exact mechanisms and implications of NETs in TB, SARC, and other diseases with granulomas such as AP are still an area of active research, and their precise role in disease progression and pathology is not yet fully understood. The balance between NET formation and degradation needs to be strictly maintained, as detrimental effects of NETs emerge during the process of NET ripening. The above-mentioned studies have already investigated the role of neutrophils and NETs in TB. However, the diagnostic significance of neutrophil activation and NET markers in different subtypes of TB still remains elusive.

As in several conditions of induction and resolution of inflammation, there are limitations of our study. An important question remains unanswered: which came first, the chicken or the egg? Do granulomas caseate due to the action of NETs or do granulomas attract neutrophils and then induce the formation of NETs? Furthermore, a correlation of circulatory NET degradation products pre-relapse to relapse has not been performed in the same patients, as no sera were available for the pre-relapse phase.

Here we examined NET formation and NE enzymatic activity in various types of clinical TB. Since NET formation was increased in patients in relapse, and those with extensive tissue damage, we propose NETs ELISA for the screening of patients at a heightened risk of relapse and discuss the importance of balancing NET formation to prevent tissue damage or even relapse. By analyzing tissue NET formation in granulomas in lung and lymph node biopsies from TB and SARC patients, we report tissue NET formation as a common feature of caseating granulomas. Moreover, granulomas are filled with DNase-resistant Z-form DNA, which is most abundant in the non-caseating granulomas found in SARC lymph nodes. Lastly, many pulmonary vessels in TB are occluded and filled with neutrophils, contributing to disease pathology.

### Supplementary information

Supplemental Material

## Data Availability

Data generated during the study are within the article or Supplementary Materials.

## Code availability

We processed and further analyzed the images by morphometry using Photoshop CC 2018 (Adobe, Munich, Germany). Then we extracted and exported the mean fluorescence intensity (MFI) as an R data table object. We further performed FIt-SNE dimensionality reduction using the function ‘run.fitsne()’ from the Spectre R package (10.1002/cyto.a.24350). Instructions and the source code, which was accessed on 16 November 2023, are provided at https://github.com/ImmuneDynamics/spectre.
