# Supplementary material for: Neutrophil extracellular traps characterize caseating granulomas
Source: Cell Death Dis. 2024 Jul 31;15(7):548. doi: 10.1038/s41419-024-06892-3 (PMC11291884; doi:10.1038/s41419-024-06892-3)
Supplement: Supplementary file 1 — Supplemental Material [file 41419_2024_6892_MOESM1_ESM.docx]

**Neutrophil extracellular traps characterize caseating granulomas**

Leticija Zlatar^1,2,*,†^, Jasmin Knopf^1,2,3^, Jeeshan Singh^1,2^, Han Wang^1,2^, Marco Muñoz-Becerra^1,2^, Irmgard Herrmann^1,2^, Rebecca C. Chukwuanukwu^1,4^, Markus Eckstein^5^, Philip Eichhorn^5^, Ralf J. Rieker^5^, Elisabeth Naschberger^5,6^, Andreas Burkovski^7^, Veit Krenn^8^, Rostyslav Bilyy^9^, Tetiana Butova^10^, Iryna Liskina^11^, Ihor Kalabukha^12^, Oleg Khmel^12^, Michael Boettcher^3^, Georg Schett^1,2^, Dmytro Butov^13,$^, Anton Tkachenko^14,15,$^, and Martin Herrmann^1,2,3,16,$^

^1^ Department of Internal Medicine 3 – Rheumatology and Immunology, Friedrich-Alexander-University Erlangen-Nürnberg and Universitätsklinikum Erlangen, Erlangen, Germany.

^2^ Deutsches Zentrum für Immuntherapie (DZI), Friedrich-Alexander-University Erlangen-Nürnberg and Universitätsklinikum Erlangen, Erlangen, Germany.

^3^ Department of Pediatric Surgery, University Medical Center Mannheim, University of Heidelberg, Mannheim, Germany.

^4^ Immunology Unit, Medical Laboratory Science Department, Faculty of Health Sciences, Nnamdi Azikiwe University, Awka, Nigeria.

^5^ CCC Comprehensive Cancer Center (CCC) Erlangen and Institute of Pathology, Friedrich-Alexander-University Erlangen-Nürnberg, Erlangen, Germany.

^6^ Division of Molecular and Experimental Surgery, Universitätsklinikum Erlangen, Friedrich-Alexander Universtität Erlangen-Nürnberg, Erlangen, Germany.

^7^ Microbiology Division, Department of Biology, Friedrich-Alexander-University Erlangen-Nürnberg, Erlangen, Germany.

^8^ MVZ-Center for Histology, Cytology and Molecular Diagnostics, Trier, Germany.

^9^ Lectinotest R&D, Lviv, 79000, Ukraine.

^10^ Outpatient department, Merefa District Hospital, Merefa, 62472, Ukraine.

^11^ Department of pathomorphology, State Organization "National Institute of Phthisiology and Pulmonology named after F.G. Yanovsky of the National Academy of Medical Sciences of Ukraine", Kyiv, 03680, Ukraine.

^12^ Department of surgical treatment of tuberculosis and non-specific lung diseases, State Organization "National Institute of Phthisiology and Pulmonology named after F.G. Yanovsky of the National Academy of Medical Sciences of Ukraine", Kyiv, 03680, Ukraine.

^13^ Department of Infectious diseases and Phthisiology, Kharkiv National Medical University, 4 Nauky Avenue, Kharkiv, 61022, Ukraine.

^14^ Research Institute of Experimental and Clinical Medicine, Kharkiv National Medical University, Kharkiv, 61022, Ukraine.

^15^ BIOCEV, First Faculty of Medicine, Charles University, Prumyslova 595, 25250 Vestec, Czech Republic.

^16^ FAU Profile Center Immunomedicine (FAU I-MED), Friedrich-Alexander-Universität (FAU) Erlangen-Nürnberg, Schlossplatz 1, D-91054 Erlangen, Germany.

^$^D. B., A.T., and M.H. equally contributed to senior authorship.

^*^ Correspondence: Leticija.Zlatar@uk-erlangen.de; Tel.: +49-9131-85-34788

^†^ This manuscript will be used as part of Leticija Zlatar’s doctoral thesis.

**
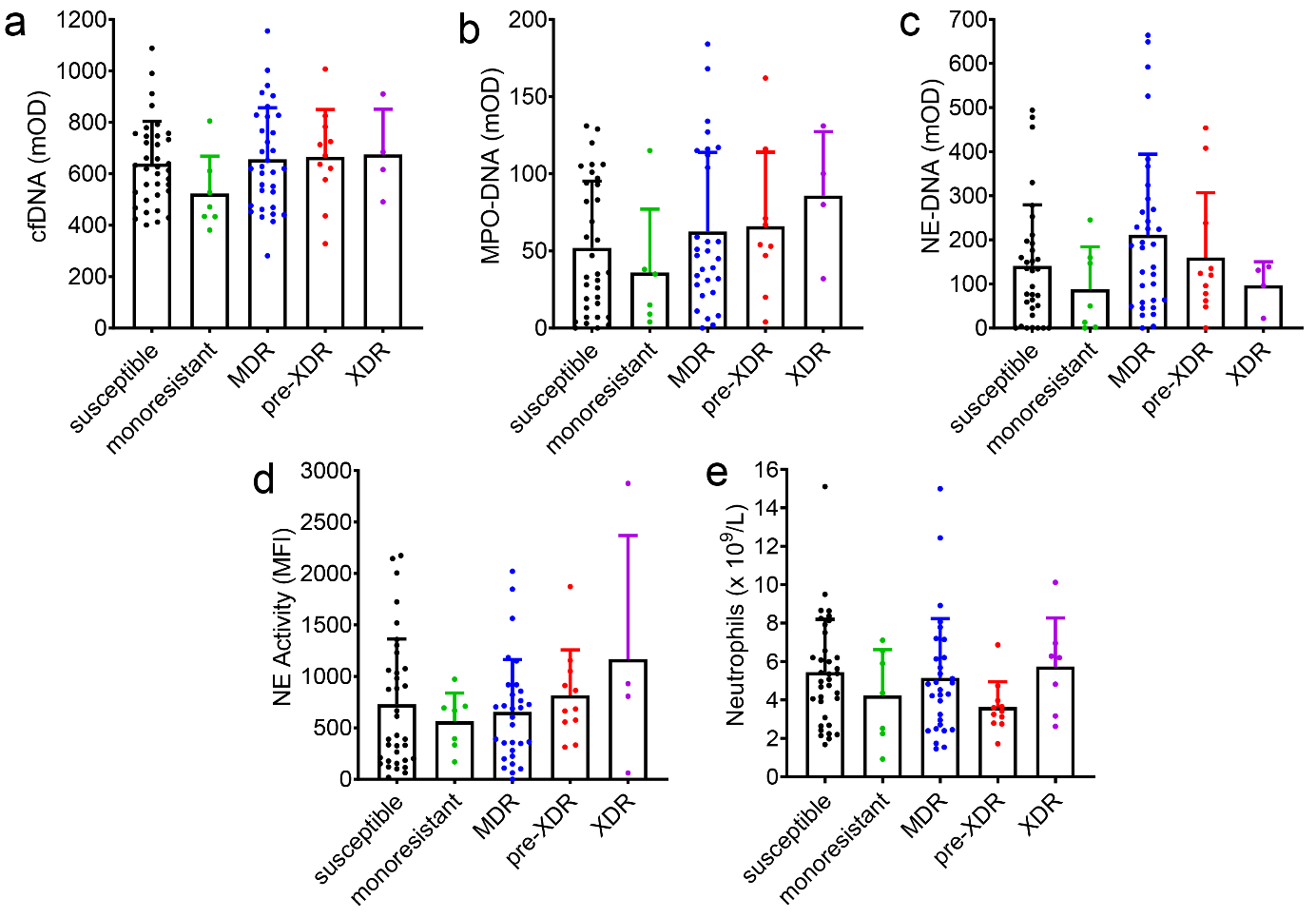
Figure S1.** **NET degradation products can be detected among various types of resistant TB.**

Markers for NETs in the sera of patients with TB grouped by the type of resistant TB. We quantified **a** cfDNA in the sera by PicoGreen fluorescence, **b** MPO‑DNA and **c** NE-DNA complexes by ELISA, **d** NE activity by the conversion of a specific fluorogenic substrate and **e** neutrophil levels by by leukogram profiling. Note that there are no significant differences in NET formation, NE activity or neutrophil counts between various TB subtypes. Statistical analyses were performed employing the **a** ordinary one-way ANOVA, **b‑d** Kruskal‑Wallis test or **e** Mann-Whitney test. Data are presented as mean ± standard deviation (SD). Abbreviations: cfDNA, cell-free DNA; MDR, multi-drug resistant; pre-XDR, pre-extensively drug resistant; XDR, extensively drug resistant; MPO, myeloperoxidase; NE, neutrophil elastase; MFI, mean fluorescence intensity.

**
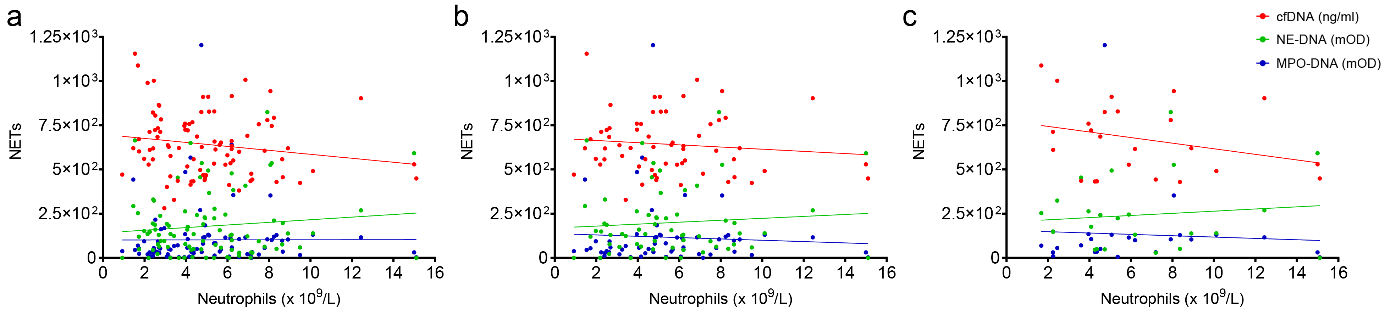
Figure S2. NET formation in TB patients reflects neutrophil activation state rather than neutrophil counts.**

Correlation analyses between NETs and neutrophil counts in the sera of TB patients. **a** all TB patients (*n* = 91), **b** TB patients with extensive tissue destruction (*n* = 61) and **c** TB patients in relapse (*n* = 24) employing simple linear regression. Note that there is no significant correlation between the two variables in any of the cohorts. Abbreviations: NETs, neutrophil extracellular traps; cfDNA, cell-free DNA.

**
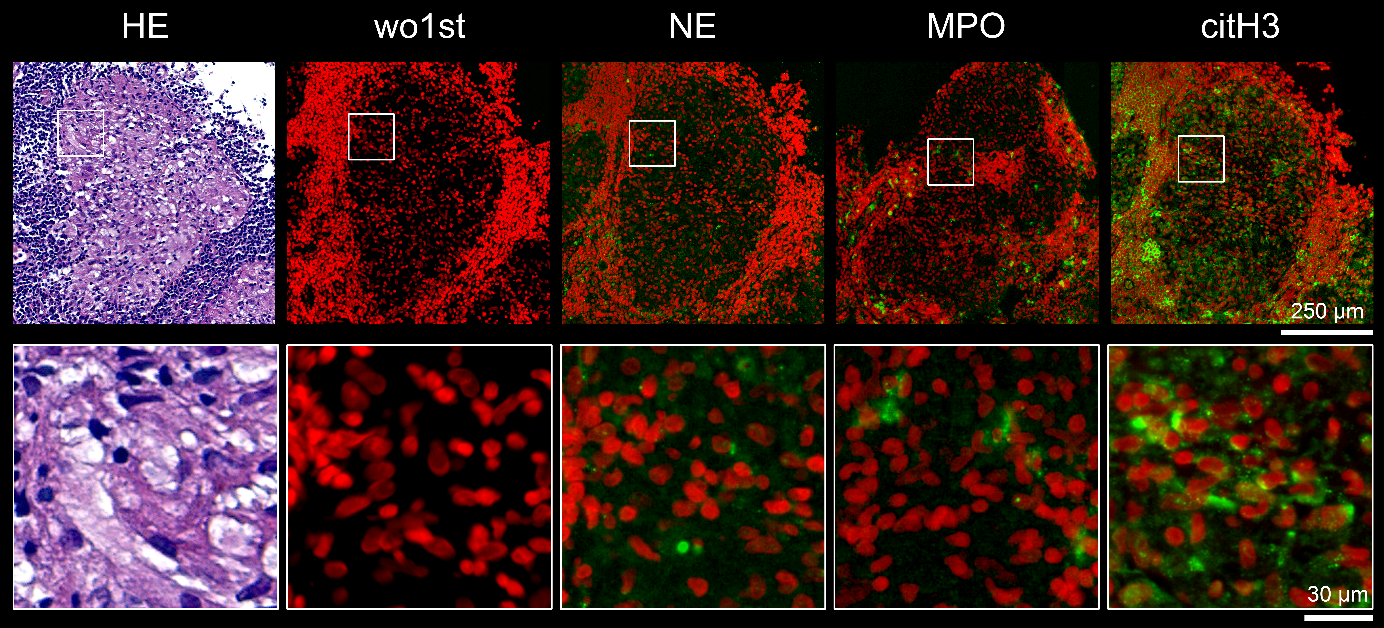
Figure S3. Non-caseating granulomas are a poor source of NETs.**

Comparison of various NET staining (NE, MPO, citH3) in a representative lymph node granuloma from SARC patient. Upper panel displays the whole granuloma with white squares marking regions which were enlarged for better visibility and are displayed in the lower panel. All antigens are shown in green, and sorted according to their abundance from left to right (low to high). Hoechst33342 was used as a DNA dye (red). Mock staining without the primary antibody (wo1st) served as control. HE staining, purple.

**
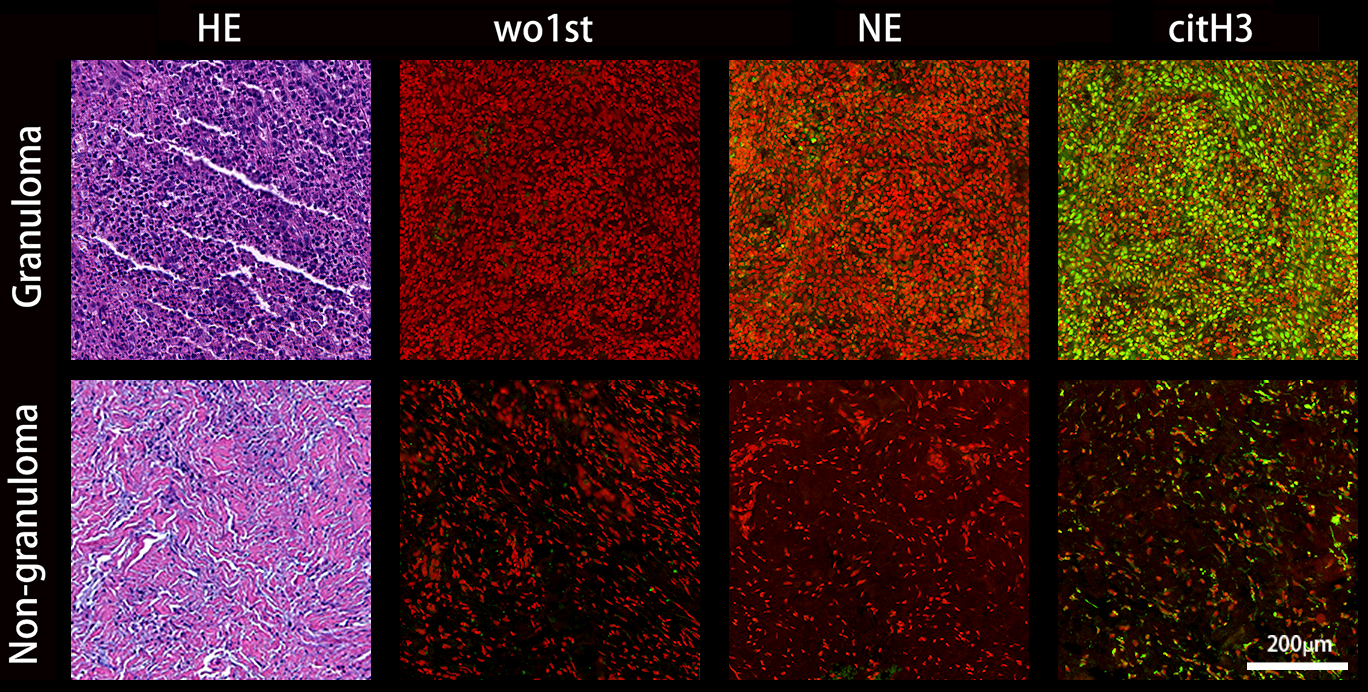
Figure S4. Neutrophils infiltrate the apical periodontitis granuloma.**

The top row displays NE (green) and citH3 (green) signals in apical periodontitis (AP) granuloma. The bottom row shows NE (green) and citH3 (green) staining in non-granulomatous AP. Red represents DAPI, indicating DNA. Wo1st served as a negative control. The first column represents HE staining, appearing in purple.

**
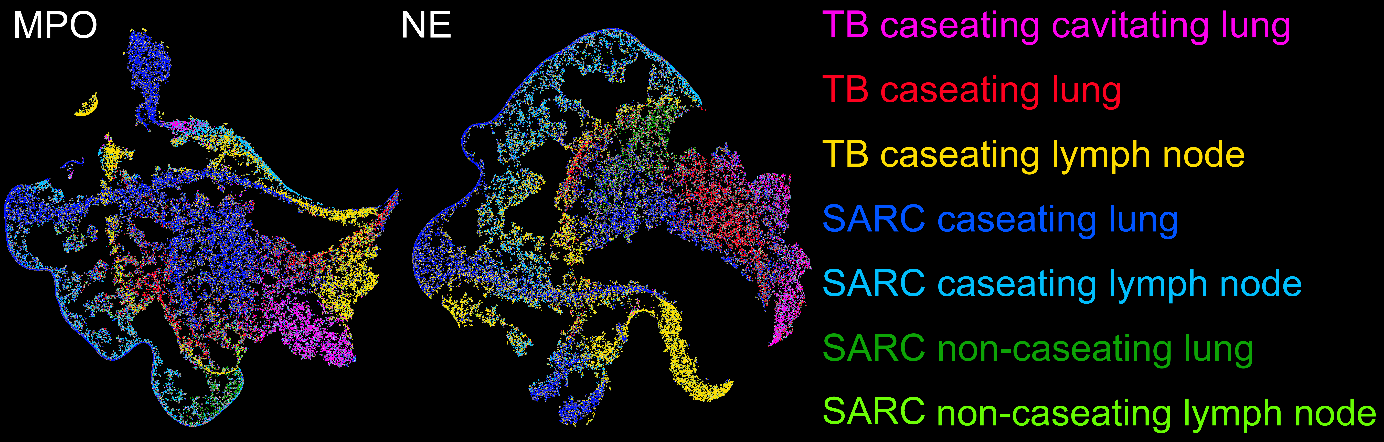
Figure S5. Granulomas are heterogenous in staining for NET markers.**

FIt-SNE plots obtained by morphometric analysis of MPO or NE MFI. Differentiation is based on disease, granuloma, and organ. Abbreviations: TB, tuberculosis; SARC, sarcoidosis.

**Table S1. Antigens detected by immunohistological analyses of tissue specimens from TB and SARC patients - granuloma vs. intergranulomatous tissue ^1^.**

| **TUBERCULOSIS** | | |
| --- | --- | --- |
| ***Lungs*** | ***Granuloma*** | ***Intergranulomatous tissue*** |
| NE (75%) | 0.00 | 0.75 |
| citH3 (75%) | 0.33 | 0.75 |
| MPO (75%) | 0.33 | 0.75 |
| ***Lymph nodes*** | ***Granuloma*** | ***Intergranulomatous tissue*** |
| NE (67%) | 1.50 | 1.00 |
| citH3 (100%) | 2.00 | 1.67 |
| MPO (67%) | 2.50 | 2.00 |
| **SARCOIDOSIS** | | |
| ***Lungs*** | ***Granuloma*** | ***Intergranulomatous tissue*** |
| NE (83%) | 1.00 | 0.83 |
| MPO (100%) | 1.33 | 0.83 |
| citH3 (83%) | 1.50 | 0.50 |
| ***Lymph nodes*** | ***Granuloma*** | ***Intergranulomatous tissue*** |
| NE (50%) | 0.17 | 1.50 |
| MPO (50%) | 0.67 | 0.25 |
| citH3 (100%) | 2.00 | 0.25 |
| ^1^ All staining were visually estimated for each sample (*n* = 7 for TB, *n* = 12 for SARC), and a mean score ranging 0-3 was calculated (0: green, no antigen detected; 3: red, high antigen prevalence & signal intensity). Parentheses contain the percentage of samples in which the relevant antigen was detected. | | |
